# Proceedings of the Dengue Symposium at the 9th International Congress of Tropical Pediatrics

**DOI:** 10.1179/2046904712Z.00000000044

**Published:** 2012-05

**Authors:** Usa Thisyakorn, Krisana Pengsaa

**Affiliations:** 1 Faculty of Medicine, Chulalongkorn University; 2 Faculty of Tropical Medicine, Mahidol University, Bangkok, Thailand

Dengue is the most important mosquito-borne viral disease. With four different serotypes, it is a burden throughout tropical and subtropical regions and a potential threat to almost half of the world’s population. Recent studies estimate that 50–100 million people are infected each year, of whom about 500,000 develop dengue haemorrhagic fever – a severe form of the disease – and 22,000 of whom die. Dengue haemorrhagic fever is also a leading cause of hospitalisation, placing tremendous pressure on strained medical resources, with an associated major economic and societal impact. Many factors have contributed to the recent dramatic rise in the number of cases of dengue fever, including increased urbanisation and travel. The heavy burden of dengue can only be lifted by strong cooperation between international institutions and agencies to create a new strategy targeting prevention of deaths, reducing morbidity and lessening the associated social and economic losses. In addition, a dengue vaccine is seen as the best way to control dengue effectively. Proper management of dengue infection, mosquito control measures as well as a candidate dengue vaccine, requires a comprehensive update of knowledge in all relevant aspects.

This parallel symposium was organised by the Thailand Chapter of the International Society of Tropical Pediatrics on 20 October 2011, in collaboration with the 9th International Congress of Tropical Pediatrics, which was held on 18–20 October 2011 in Bangkok, Thailand. The objective of the symposium was to provide the participants with a unique opportunity to share their experiences and discuss various issues relating to the pathophysiology, epidemiology, management and control of dengue. Through this symposium, participants gained knowledge and experience from invited speakers from various parts of highly endemic areas. In this context, the symposium also illustrated the importance of sharing best practice and knowledge across all regions and specialities involved in the struggle against dengue. These proceedings summarise the Parallel Symposium on Dengue held at the Queen Sirikit National Convention Center, Bangkok, Thailand, on 20 October 2011.

The supplement begins with an interesting commentary from Scott Halstead as he seeks to analyse and resolve six key controversies in dengue fever research, including the utility of the 1997 World Health Organization (WHO) case definition of dengue haemorrhagic fever and an examination of several theories of dengue infection pathogenesis. Following this, Roberto Tapia-Conyer and colleagues consider the more practical aspects of dengue fever management: community participation, structured by an integrated management strategy. It has the overall aim of improving the efficacy, cost-effectiveness, environmental impact and sustainability of vector control strategies. Tapia-Conyer and his co-authors then discuss in detail the problem of dengue fever infection in Latin America overall. They suggest various ways in which the disease could be combated, including using a vaccine or involving the community, but conclude that politics, finance and co-operation are all areas that need to be managed. A contrasting case study is then presented by Fred Were, focusing on dengue in Africa and the possible differences in the spread of the disease compared with the remainder of the dengue-endemic population. However, Were points out that it is difficult to characterise the disease in Africa owing to the poor surveillance infrastructure and under-recognition of the disease, as there is often a greater focus on malaria. Terapong Tantawichien follows this with a discussion of the increasing prevalence of dengue infection in adolescents and adults, with an in-depth analysis of its clinical manifestations and specific treatment in this age group. On a similar theme, Annelies Wilder-Smith focuses on a particular subset of the adult population threatened by dengue infection – travellers. Travellers are not only at risk themselves but they contribute to the spread of dengue; furthermore, they may serve to alert the international community to current epidemics. Sri Rezeki Hadinegoro considers the diagnoses of dengue fever, dengue haemorrhagic fever and dengue shock syndrome in relation to the WHO classification systems of 1997 and 2009. There has been much debate surrounding these classification criteria and Hadinegoro suggests that the evolution of dengue over past years has contributed to the limited applicability of the older case definitions. Following the DENCO study, the newer classification system was formed, but the effectiveness of this is still being debated. Finally, Nguyen Thanh Hung describes the types of intravenous solutions for treatment of children with dengue; he concludes that isotonic crystalloid solutions can be used for the majority of children, and colloids can be considered a second option.

We should like to express our sincere thanks to all of the distinguished invited speakers and resource persons for their contribution. We hope that these proceedings will prove of great interest, and help the global dengue community to improve the prevention, diagnosis and management of dengue infection.

